# Blockade of IL-36 Receptor Signaling Does Not Prevent from TNF-Induced Arthritis

**DOI:** 10.1371/journal.pone.0101954

**Published:** 2014-08-11

**Authors:** Anja Derer, Bettina Groetsch, Ulrike Harre, Christina Böhm, Jennifer Towne, Georg Schett, Silke Frey, Axel J. Hueber

**Affiliations:** 1 Department of Internal Medicine 3 and Institute for Clinical Immunology, University Hospital Erlangen, Erlangen, Germany; 2 Department of Radiation Oncology, University Hospital Erlangen, Erlangen, Germany; 3 Department of Inflammation Research, Amgen Inc., Longmont, Colorado, United States of America; French National Centre for Scientific Research, France

## Abstract

**Introduction:**

Interleukin (IL)-36α is a newly described member of the IL-1 cytokine family with a known inflammatory and pathogenic function in psoriasis. Recently, we could demonstrate that the receptor (IL-36R), its ligand IL-36α and its antagonist IL-36Ra are expressed in synovial tissue of arthritis patients. Furthermore, IL-36α induces MAP-kinase and NFκB signaling in human synovial fibroblasts with subsequent expression and secretion of pro-inflammatory cytokines.

**Methods:**

To understand the pathomechanism of IL-36 dependent inflammation, we investigated the biological impact of IL-36α signaling in the *hTNF*tg mouse. Also the impact on osteoclastogenesis by IL-36α was tested in murine and human osteoclast assays.

**Results:**

Diseased mice showed an increased expression of IL-36R and IL-36α in inflamed knee joints compared to wildtype controls. However, preventively treating mice with an IL-36R blocking antibody led to no changes in clinical onset and pattern of disease. Furthermore, blockade of IL-36 signaling did not change histological signs of TNF-induced arthritis. Additionally, no alteration on bone homeostasis was observed in ex vivo murine and human osteoclast differentiation assays.

**Conclusion:**

Thus we conclude that IL-36α does not affect the development of inflammatory arthritis.

## Introduction

New members of the IL-1 family cluster have recently been identified consisting of IL-1F6 (IL-36α), IL-1F8 (IL-36β), IL-1F9 (IL-36γ) and the receptor antagonist IL-1F5 (IL-36Ra). They display sequence homology with the IL-1 members IL-1α, IL-1β and IL-18 of 21–37%. IL-36Ra has also a 52% similarity with IL-1Ra [1]. These ligands bind to the same receptor, the IL-36 receptor (IL-36R), which subsequently recruits the IL-1 receptor accessory protein (IL-1RAcP) forming a heterodimeric signaling complex, the latter is also used by IL-1α and IL-1β. The IL-36 cytokines are expressed in internal epithelial tissues and in areas, which are exposed to pathogens. However, the exact cellular source(s) of the individual ligands are not clearly defined yet. Nevertheless, various analyses suggested their implication in inflammatory diseases. So far, the role of IL-36 family members was mainly studied in psoriasis (reviewed in [2]) in which inflamed skin expresses increased IL-36R, IL-36α and IL-36Ra [3]. The functional deletion of the antagonist leads to severe generalized psoriasis in humans [4]. This inflammatory phenotype was also seen in murine models overexpressing IL-36α in basal keratinocytes which develop age-dependent inflammatory skin lesions with features of human psoriasis like acanthosis, hyperkeratosis, inflammatory cell infiltrates and enhanced cytokine production [3]. In this mouse model, IL-36α enhances the production of pro-inflammatory cytokines IL-17A, IL-23p19 and TNF-α in skin inflammation, cytokines which are also involved in rheumatoid arthritis [5]. The blockade of TNF-α or IL-23p19 diminished clinical signs like epidermal thickness, inflammation and cytokine production. In addition, an induction of IL-36 cytokines in IL-17A and TNF-α stimulated primary human keratinocytes as well as a correlation of gene expression of IL-36 with Th17 cytokines was seen in lesions of psoriasis patients [6]. On a cellular basis, IL-36R is also expressed on murine bone-marrow derived dendritic cells (BMDCs) and CD4^+^ T cells. Stimulation with its ligands induced the production of IL-12, IL-1β, IL-6, TNF-α and IL-23 in BMDCs, while stimulated CD4+ T cells produced IFN-γ, IL-4 and IL-17 [7].

Further it has been shown that TNF-α is induced by and can induce IL-36α which contributes to the pro-inflammatory function of IL-36α [5]. Focusing on autoimmune arthritis IL-36β (IL-1F8), another family member, induced the expression of IL-6, IL-8 and nitric oxygen (NO) in primary synovial fibroblasts; however, the level of IL-36β in serum and synovial fluid of patients with rheumatoid arthritis, osteoarthritis and septic shock did not correlate with the grade of inflammation [8]. We could demonstrate that the receptor as well as IL-36α and its antagonist IL-36Ra are expressed in synovial tissue of arthritis patients, suggesting a potential role in inflammatory arthritis [9].

Therefore, we sought to investigate the physiological relevance of these findings in a TNF-α driven animal model of arthritis, the *hTNF*tg mouse. Further, we tested the biological effect of IL-36α stimulation on human and murine osteoclastogenesis.

## Material and Methods

### Mice treatment and clinical assessment

Mice experiments were approved by the government of Mittelfranken (Regierung von Mittelfranken, Regierungsbescheid, 54-2532.1-26/12) and mice were housed in the animal facility (Franz-Pentzold-Zentrum) of the University of Erlangen-Nuremberg. The heterozygous Tg197 TNF-transgenic mice (C57BL/6, hTNFtg) used in the present study have been described previously and were kindly provided by Prof. G. Kollias (Fleming Research Institute, Vari, Greece) [10]. Arthritis and bone changes in this model have been shown to be altered by multiple approaches. The arthritis phenotype in these mice was shown to be suppressed by treatment with TNF inhibitors. Furthermore inflammation-induced bone destruction was suppressed by anti-IL-17, IL-6R blockade and CTLA-4 [11]–[17]. Mice treatment and clinical evaluation was performed weekly, starting four weeks after birth. Male hTNFtg (genetic background C57BL/6) mice were injected with 150 µg (in 200 µl) of a blocking antibody against IL-36R (M616, Amgen) i.p.; the procedure was repeated every third day for a period of four weeks. Sex and age matched PBS injected hTNFtg mice severed as controls. Arthritis was evaluated in a blinded manner as previously described [18]. Briefly, we determined the course of the disease by assessing four clinical parameters: weight, grip strength, paw swelling (metatarsal joints) and joint swelling (ankle joint). The grip strength was examined by using a 3-mm-diameter wire, and was scored on a scale of 0 to −4 (0 = normal grip strength, −1 = mildly reduced, −2 = moderately reduced, −3 = severely reduced, −4 = no grip strength). In addition, the paw swelling was examined in both hind paws by measuring the paw and the joint thickness with a caliper. Mice were killed by cervical dislocation at 8 weeks of age. Due to the results presented below we omitted an isotype controlled experiment.

### Bone histomorphometry

Hind paws were fixed overnight in 4% formalin and then decalcified in 14% EDTA (Sigma) at 4°C until the bones were pliable. Serial paraffin sections (2 µm) from all paws were stained with hematoxylin and eosin (H&E) for assessment of synovial inflammation and toluidine blue (TB) for proteoglycan loss in the articular cartilage. Tartrate-resistant acid phosphatase (TRAP) staining was performed by using a leukocyte acid phosphatase staining kit (Sigma) for detection of osteoclasts and bone erosions in the tarsal joints. Synovial inflammation, cartilage loss, bone erosions and osteoclast numbers were quantified with the use of a Zeiss Axioskop 2 microscope (Zeiss) equipped with a digital camera and image analysis system (OsteoMeasure; OsteoMetrics, Decatur, Georgia), as described previously [16]. The synovitis extend containing infiltrated cells defined the area of inflammation that was quantified on H&E–stained sections. Total scores were calculated as the sum of the areas of inflammation per section of the metatarsal joints; two sections per mouse were investigated. Osteoclasts (≥2 nuclei; TRAP positive cells) were assessed in TRAP-stained serial sections. Erosion areas were quantified in the same sections and identified by the presence of inflamed synovial tissue within the outer cortical bone surface. Cartilage loss was assessed by quantifying the ratio between toluidine blue stained cartilage (intact cartilage) and unstained cartilage (destroyed cartilage) in all joints of the paw.

### Enzyme Linked Immunosorbent Assay (ELISA)

Serum levels of murine IL-6, KC and human TNF-α from 8-week-old mice were measured using the ELISA protocol according to the manufacturer's instructions (R&D Systems).

### Human and murine osteoclast culture

Donors gave written informed consent, and sample use for research was approved by the local Ethics committee (Ethics committee of the University Erlangen, Nr 3982). Human monocytes were purified by plastic adhesion of peripheral blood mononuclear cells that had been isolated from EDTA-blood of normal healthy donors using a Ficoll gradient (Lymphoflot, BioRad). Osteoclasts were differentiated in α-Mem (Invitrogen) containing 10% fetal bovine serum (Biochrome) and 1% penicillin/streptomycin (Invitrogen), supplemented with 30 ng/ml M-CSF, 10 ng/ml RANKL and 1 ng/ml TGF-β (all PeproTech) in the presence of IL-36α(truncated, R&D systems), IL-36Ra or the soluble IL-36 receptor (R&D systems) in the indicated amounts. For murine osteoclast assay, bone marrow from C57BL/6 mice was isolated and cultivated over night at 37°C and 5% CO_2_ in α-Mem (Invitrogen) containing 10% fetal bovine serum (Biochrome) and 1% penicillin/streptomycin (Invitrogen). Osteoclasts were differentiated by stimulation with 30 ng/ml M-CSF, 10 ng/ml RANKL and additional 100 ng/ml recombinant mouse IL-36α. Osteoclast differentiation was evaluated by staining for TRAP using a Leukocyte Acid Phosphatase Kit (Sigma) according to the manufacturer's instructions.

### Quantitative Real-time PCR

mRNA expression during human and murine osteoclastogenesis as well as in the knee joints from wildtype and *hTNF*tg mice was assessed via quantitative real-time PCR. mRNA expression of *hCtsK* (5′-AGAAGACCCACAGGAAGCAA-3′ and 5′-GCCTCAAGGTTATGGATGGA-3′), *hIL-1Rrp2* (*IL-36R*) (5′-CTGGACAAGCCGTGGCCAATGT-3′ and 5′-AGCCCAGCGATTCGGGGACC-3′), *hIL-1RAcP* (5′-ACCTCTGAGGATCTCAAGCGCAGC-3′ and 5′-TGCTTCACCTTGGCTGCTTTGGC-3′), *hHsp90ab1* (5′-GCGCAGTGTTGGGACTGTCTGG-3′ and 5′-TCCTCCTCTCCATGGTGCACTTCC-3′), *mTRAP* (5′-CACTCCCACCCTGAGATTTGT-3′ and 5′-CATCGTCTGCACGGTTCTG-3′), *mCtsK* (5′-GGCCAGTGTGGTTCCTGTT-3′and 5′-CAGTGGTCATATAGCCGCCTC-3′), *mIL-1Rrp2* (5′- TGCTTCTGCTTTTCGTGGCAGCA-3′and 5′- GCCCCGTTTGTTTCTGGCGG-3′), *mIL-36α* (5′- ACACATTGCTCTGTGGCACT-3′ and 5′- GGAGGGCTCAGCTTTCTTTT-3′), and *mβ-actin* (5′- TGTCCACCTTCCAGCAGATGT-3′and 5′- AGCTCAGTAACAGTCCGCCTAGA-3′) was measured by real time PCR with Hsp90ab1 as control for human samples and β-actin as control for murine samples (all Life Technologies).

### Statistical analyses

All statistical analyses were performed using GraphPad Prism software (GraphPad software, Inc.) with Student's t-test. Data are represented as mean ±SEM. * p<0.05, ** p<0.01, *** p<0.001.

## Results

### IL-36R and IL-36α are expressed in joints of hTNFtg mice

**Figure 1:**
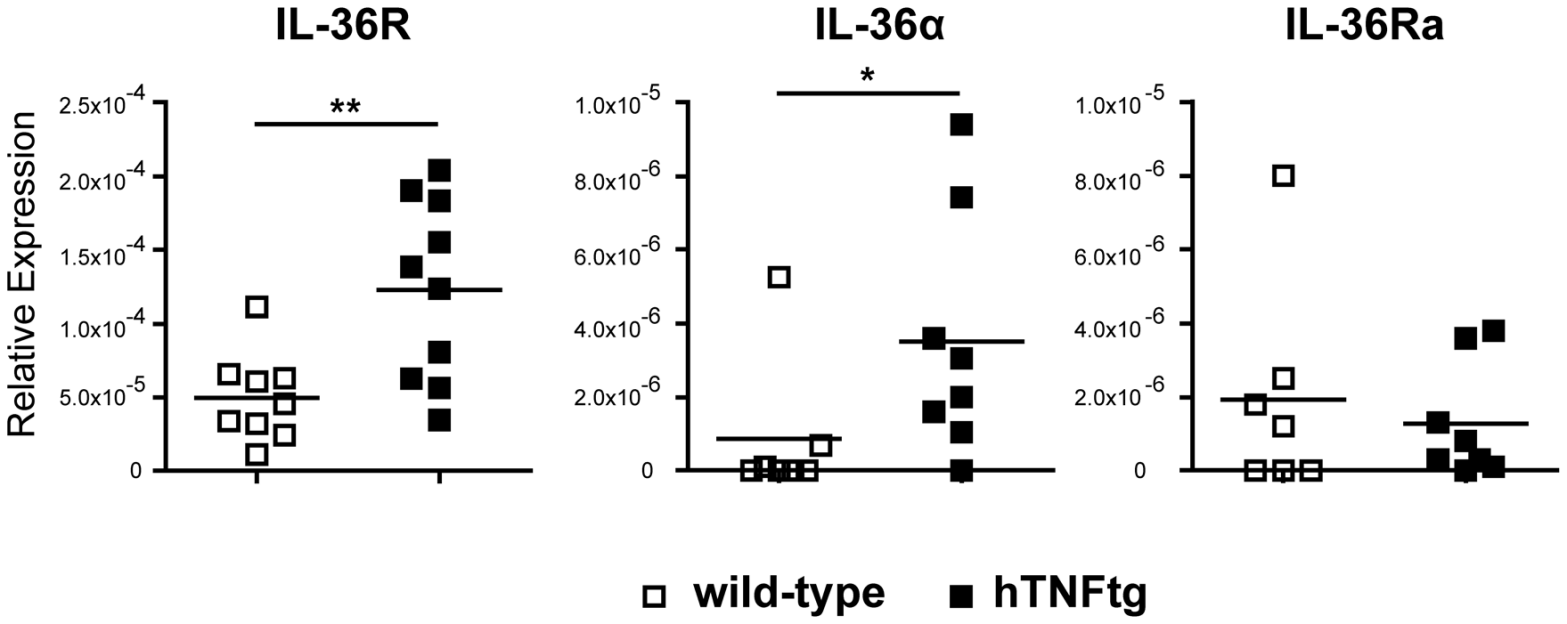
Increased expression of IL36R and IL36α in hTNFtg mice. otal knee RNA from knee-joints of 6-week-old male wild-type and hTNFtg mice was isolated and quantitative real-time PCR for IL-36 family members was performed. (A) IL36R, (B) IL-36α and (C) IL-36Ra. Relative Expression was calculated from the ratio of the gene of interest to the housekeeping gene β-actin (n = 7–10). Graphs depict mean ±SEM. *p≤0,05, **p≤0,01, ***p≤0,001.

The *hTNF*tg mouse is a commonly used model for rheumatoid arthritis which develops clinical symptoms of arthritis spontaneously at 5 to 6 weeks after birth by constitutively expressing the human TNF gene [10]. Initially, we examined the expression of IL-36R, IL-36α and IL-36Ra mRNA in normal (wildtype) and inflamed (*hTNF*tg) knee joints using quantitative real-time PCR. For IL-36R, basal expression could be detected in all joints investigated but was significantly enhanced in the joints of *hTNF*tg mice (Figure 1A). The expression of its ligand IL-36α could be detected in inflamed *hTNF*tg joints; however, it was hardly detectable in wildtype knee joints (Figure 1B). Its antagonist IL-36Ra was expressed on comparable levels in both wildtype and inflamed joints (Figure 1C).

### Blockade of IL-36 signaling does not influence the clinical course of TNF-induced arthritis

**Figure 2:**
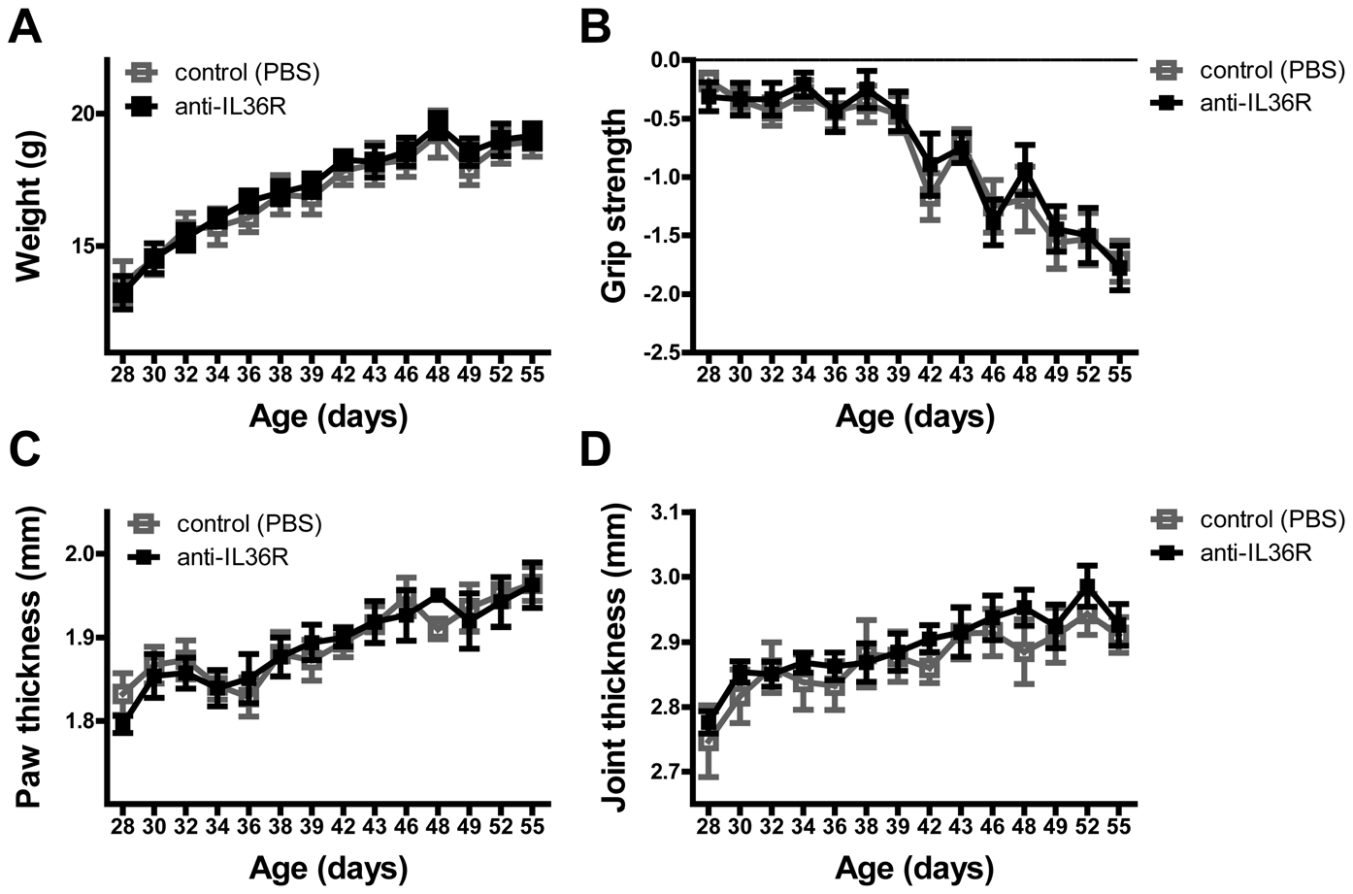
Unaltered clinical signs of arthritis in anti-IL36R treated hTNFtg mice. Clinical parameters: (A) body weight, (B) grip strength, (C) paw thickness and (D) joint thickness were regularly assessed in anti-IL36R treated hTNFtg mice between 4 and 8 weeks of age. Values represent the mean ±SEM (PBS control group n = 8; treatment group n = 9).

According to the enhanced expression of IL-36R and IL-36α in inflamed joints we hypothesized that IL-36R signaling blockade could ameliorate disease onset as well as severity. Therefore, we used an antibody against the IL-36 receptor that has been shown to actively suppress the psoriasis phenotype in IL-36tg [5]. Using anti-IL-36R antibody or PBS, *hTNF*tg mice were treated from 4 to 8 weeks of age, beginning at a time point where the clinical onset is not detectable yet. The assessment of clinical parameters revealed no significant differences in the development of weight of the treated mice compared to the control group (Figure 2A). In addition, the loss of grip strength appeared similar in both groups (Figure 2B). This clinical parameter was further confirmed by the measurement of metatarsal as well as ankle joint swelling (Figure 2C and D), whereby the course of the increase in thickness was analogous in the treatment- and the control-group. In conclusion, we did not observe differences in the severity of the disease course.

### Blockade of IL-36 signaling does not improve the outcome of TNF-induced arthritis

**Figure 3:**
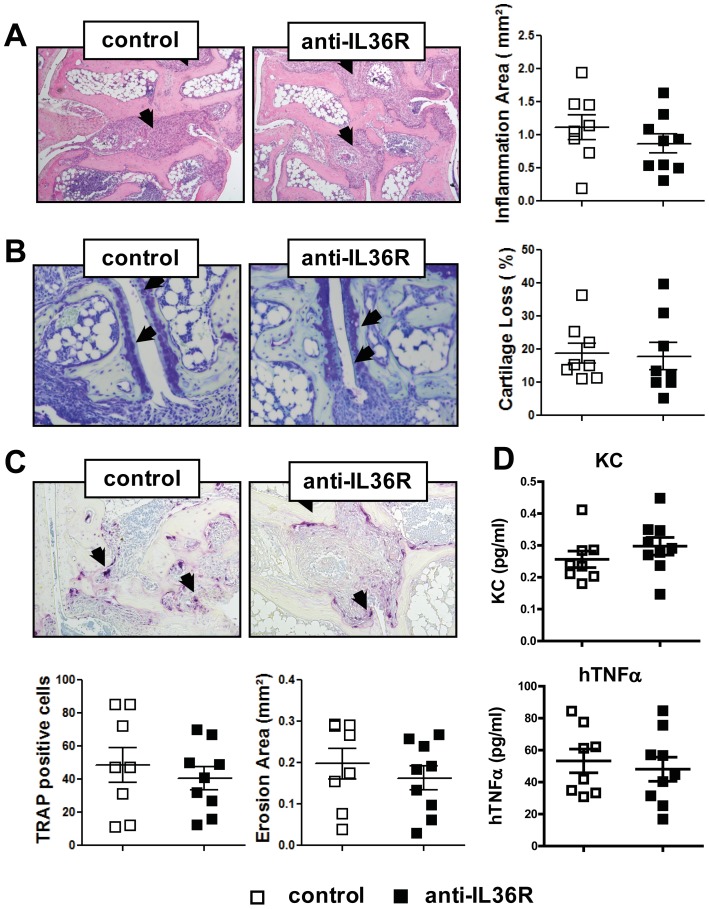
Blockade of IL-36 signaling in vivo does not influence TNF-induced arthritis. Representative pictures and histological analyses of the hind paws of anti-IL36R and control-treated mice from (A) Hematoxylin and Eosin (H&E) stainings, inflammatory areas are indicated by the arrows; (B) Toluidinblue (TB) stainings, proteoglykan loss is indicated by arrows. (C) TRAP staining of osteoclasts. (D) Sera analysis for IL-8 and the transgene TNFα of the 8-week-old mice. Graphs represent the mean ± SEM (n = 8–9).

To further investigate the effect of blocking IL-36 signaling *in vivo*, we performed histomorphometric analyses of the metatarsal joints from the hind paws of these mice. In line with the assessment of the clinical parameters, we could not detect significant changes in the area of inflammation between treatment and control group (Figure 3A). Furthermore, numbers of osteoclasts, which were recruited to the sites of inflammation as well as the area of destroyed bone (bone erosion (mm^2^)) were found to be similar in both groups (Figure 3C). Also, the analysis of cartilage erosion revealed no significant differences between both groups (Figure 3B). To determine the systemic inflammatory status, we collected the serum of the mice at 8 weeks of age and investigated levels of the pro-inflammatory cytokines IL-6, KC and hTNFα by ELISA. While IL-6 was not detectable, no differences in KC and hTNFα levels could be detected between control and treatment group, which is in accordance with our clinical and histomorphometric data (Figure 3D).

### Blockade of IL-36 signaling does not alter the systemic bone mass of hTNFtg mice

**Figure 4:**
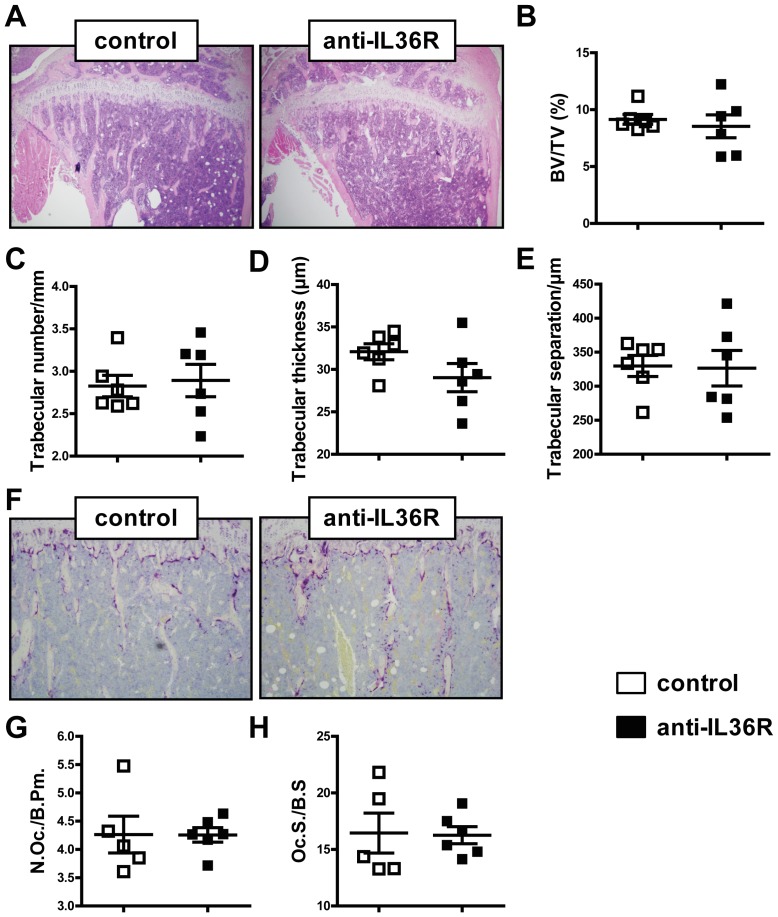
Bone homeostasis of hTNFtg mice is not altered by blockade of IL-36 signaling in vivo. Representative pictures and histomorphometry of bone parameters of trabecular bone from tibiae of 8-week-old hTNFtg mice (n = 5–6). Histological analyses of the trabecular bone of anti-IL36R and control-treated mice from (A) Hematoxylin and Eosin (H&E) stainings, (B) quantification of BV/TV (%), (C) trabecular number/mm, (D) trabecular thickness (µm), (E) trabecular separation/µm, (F) TRAP staining of osteoclasts. (D) Number of osteoclasts per bone perimeter (N.Oc./B.Pm.) and (H) osteoclast surface per bone surface (Oc.S./B.S.). Graphs represent the mean ± SEM.

We next analyzed the systemic bone mass in the tibiae of the 8-week-old *hTNF*tg mice. Bone histomorphometry was performed that revealed no significant differences of the bone mass between treatment (anti-IL36R) and control group (Figure 4, A and B). Quantification of further common bone parameters confirmed this result with no differences in the trabecular number, trabecular thickness or trabecular separation being detected (Figure 4, C–E). In agreement with the histological bone analysis, no modifications in the number of osteoclasts was observed (Figure 4, F–H). Thus, the blockade of IL-36 signaling does not improve the pathogenic effects of TNF on bone tissue.

### Osteoclastogenesis is not influenced by IL-36α treatment

**Figure 5:**
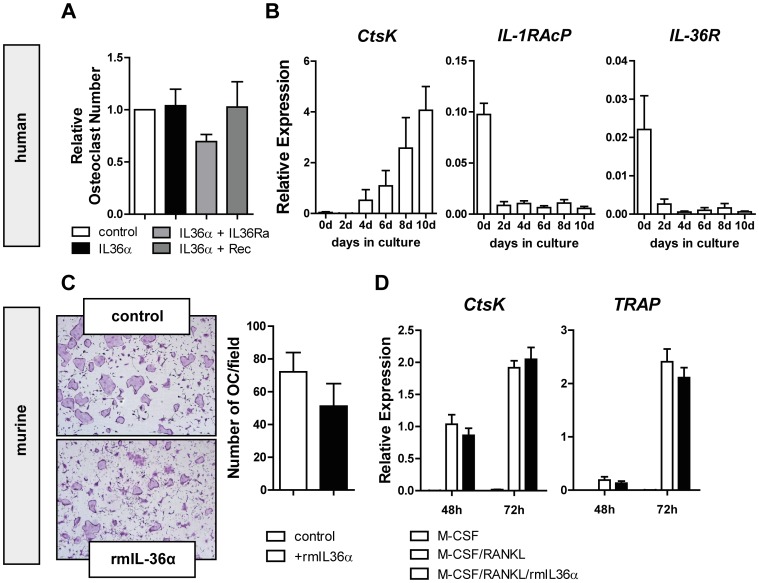
Osteoclastogenesis is not influenced by IL-36α treatment. (A) Quantification of human osteoclast assays from four different donors, done in two independent experiments; each of them was done in triplicates, treated with rhIL36α alone, rhIL36α plus IL36RA or rhIL36α plus recombinant receptor. (B) Quantitative real-time PCR for CtsK, IL-1RAcP and IL-36R of human osteoclast precursor cells stimulated with M-CSF and RANKL to achieve osteoclast differentiation. Relative Expression was calculated from the ratio of the gene of interest to the housekeeping gene Hsp90ab1. Data from three (CtsK) or four (IL-1RAcP and IL-36R) independent donors are shown. (C) Representative pictures and quantification of three independent murine osteoclast assay untreated (control) or stimulated with 100 ng/ml recombinant murine IL36α. (D) Quantitative real-time PCR for CtsK and TRAP of murine osteoclast precursor cells stimulated with M-CSF, M-CSF and RANKL or M-CSF, RANKL and rmIL-36α to achieve osteoclast differentiation. Data from three independent experiments are shown. Relative expression was calculated from the ratio of the gene of interest to the housekeeping gene β-actin. Graphs depict mean ±SEM.

To investigate the impact of IL-36 on bone homeostasis, we analyzed the effect of IL-36α on osteoclastogenesis. The stimulation of human osteoclast precursors with recombinant human IL-36α did not alter their differentiation into mature osteoclasts (Figure 5A). Additional blockade of IL-36α signaling via its antagonist IL-36Ra or the soluble IL-36 receptor did not have further impact (Figure 5A). Quantitative PCR analysis revealed a rapid down-regulation of the IL-1RAcP and the IL-36R during osteoclastogenesis (demonstrated by the up-regulation of Cathepsin K (*CtsK*) as an osteoclast-specific marker) (Figure 5B), suggesting the absence of the IL-36 receptor on mature osteoclasts. These data were confirmed in murine osteoclast assays with no influence of IL-36α on osteoclast differentiation (Figure 5C) or markers for osteoclastogenesis (Cathepsin K and TRAP, Figure 5D).

## Discussion

IL-36α and its antagonist IL-36Ra play an important role in the pathogenesis of psoriasis, an inflammatory skin disease. In our previous studies we demonstrated that IL-36α, IL-36Ra and its receptor are expressed in synovial tissue of arthritis patients [9]. These findings prompted us to investigate the function of the IL-36α signaling axis in an *in vivo*-model of arthritis to evaluate whether IL-36 family members support the pro-inflammatory cascade driving the pathogenic course of inflammatory arthritis and to determine the relevance of IL36-signaling in TNF-induced arthritis.

To classify a potential function of IL-36 in inflammatory arthritis we treated *hTNF*tg mice with a blocking monoclonal antibody against the IL-36R from 4 to 8 weeks of age [19]. However, the treatment did not modify the clinical course of arthritis in the *hTNF*tg mice (Figure 2). Furthermore, histomorphometric analyses of the metatarsal joints of the arthritic mice revealed an unaltered development of inflammation, presence of bone-resorbing osteoclasts and destruction of articular cartilage and bone in anti-IL36R treated mice. Consistent with these findings, no differences could be detected in the level of pro-inflammatory cytokines in the sera of these mice. From these data, several assumptions can be made: (i) Our data might suggest that progress and severity of inflammatory arthritis is independent of the IL-36 signaling axis. (ii) Our mouse model of arthritis is mainly driven by TNF, an extremely rigorous and strong model, which might cover potential implications of other cytokines on the development of experimental arthritis. And/or (iii) the blockade of this receptor does not only inhibit the function of IL-36 ligands but also of their antagonist; a psoriasis study by Blumberg and coworkers proposed a mechanism by which the overproduction of the ligand IL-36α together with the lack of signaling inhibition by the antagonist IL-36Ra shifts the balance between these cytokines and thereby induces the phenotype [3].

Only very recently, a paper was published by Lamacchia and coworkers, who also investigated the IL-36 receptor signaling in different models of arthritis [19]: the collagen-induced arthritis (CIA), antigen-induced arthritis (AIA) and the K/BxN serum transfer-induced arthritis model. The same monoclonal antibody (M616, Amgen) was used in the CIA and AIA model, whereas IL-36R-deficient mice were used in the AIA and serum transfer-induced arthritis. They could not detect correlations between the expression level of IL-36R, -IL-36Ra and IL-36γ and the severity of arthritis in CIA mice. Also, the treatment with the anti-IL-36R antibody did not modify the development and severity of CIA. The same was found to be true in AIA and K/BxN mice, where the arthritis course was comparable in IL-36R-deficient and wildtype mice. These results are in agreement with our data in the *hTNFtg* mouse. Thus, models of different stages of arthritis examining the role of IL-36 have now been described. Addressing the breach of tolerance, the antibody mediated effector phase and here the TNF driven cytokine/stromal interaction in joint inflammation and destruction, no arthritis model shows a potential effect by IL-36 signaling with an impact on the disease.

In addition to the inflammatory aspect of arthritis, we investigated the effect of IL-36 signaling blockade on bone homeostasis. Bone histomorphometric analyses of the tibiae of 8-week-old *hTNF*tg mice revealed no effect of the blocking antibody on the trabecular bone. Furthermore, neither the osteoclast number nor the bone surface covered by osteoclasts was changed. *In vitro* analyses showed a downregulation of the heterodimeric IL-36R during osteoclastogenesis. Human and murine osteoclastogenesis assays demonstrated that recombinant IL-36α had no impact on the cellular differentiation, suggesting that IL-36α has no relevant effect on bone resorption.

## Conclusions

In summary, we explored the physiological relevance of IL-36 signaling in the *hTNF*tg mouse. Our results indicate that the blockade of IL-36 signaling in the TNF-mouse model has no key impact on the pathogenic course of TNF-induced arthritis and therefore does not protect from TNF-induced inflammation and bone loss. Despite its pathological effect in psoriasis more information on its role in other inflammatory diseases are necessary to determine its immunological potential for disease intervention.
